# Optimizing Human Papillomavirus (HPV) Screening: Urine Sample Analysis and Associated Factors in Uzbekistan

**DOI:** 10.7759/cureus.69816

**Published:** 2024-09-20

**Authors:** Iroda P Sharipova, Ulugbek K Mirzaev, Rano I Kasimova, Yayoi Yoshinaga, Said M Shrapov, Dildora T Suyarkulova, Erkin I Musabaev

**Affiliations:** 1 Viral Infections, Scientific Research Institute of Virology, Tashkent, UZB; 2 Epidemiology, Infectious Disease Control and Prevention, Graduate School of Biomedical and Health Sciences, Hiroshima University, Hiroshima, JPN; 3 Hepatology, Scientific Research Institute of Virology, Tashkent, UZB; 4 Infectious Diseases, Central Asian University in Tashkent, Tashkent, UZB; 5 Virology Laboratory, Scientific Research Institute of Virology, Tashkent, UZB; 6 Virology, Scientific Research Institute of Virology, Tashkent, UZB

**Keywords:** hpv dna, human papillomavirus, risk factors, sensitivity, specificity, urine

## Abstract

Objective

This study assessed the accuracy of detecting Human Papillomavirus (HPV) DNA in urine samples compared to cervical samples and identified factors associated with HPV DNA positivity in Uzbekistan.

Methods

A total of 218 paired urine and cervical samples were collected from women in Uzbekistan. HPV DNA was detected using polymerase chain reaction (PCR) with genotyping. Sensitivity, specificity, positive predictive value (PPV), negative predictive value (NPV), and Cohen's Kappa coefficient were calculated. Univariate and multivariate analyses were conducted to identify factors associated with HPV DNA positivity.

Results

The study included 32.6% (71/218) positive HPV DNA by cervical samples, which demonstrated 19.7% (43/218) HPV DNA presence by urine samples. Urine HPV testing had a sensitivity of 57.7%, specificity of 98.6%, PPV of 95.3%, NPV of 82.9%, and Kappa coefficient of 60.1. To reveal factors associated with HPV DNA positivity, we set the positive 71 cases as the main group, and 147 negative samples as the control group. The results of the multivariate analysis found the association between HPV DNA positivity and included age 31-40 years (AOR=8.2), working as medical staff (AOR=7.51), condom use (AOR=0.12), having foamy (AOR=7.26) or purulent (AOR=6.84) vaginal discharge.

Conclusion

Urine HPV testing showed good agreement with cervical samples, although sensitivity was lower than some previous reports. Several sociodemographic and clinical factors were associated with HPV DNA positivity. Further optimization of urine collection, storage, and testing methods may improve sensitivity. Targeted screening of high-risk groups could enhance HPV prevention strategies in Uzbekistan.

## Introduction

Human papillomavirus (HPV) is a common sexually transmitted infection that can lead to various health issues, including genital warts and several types of cancer, most notably cervical cancer. Persistent infection with high-risk HPV types is the primary cause of cervical cancer, which remains a significant public health issue globally. The immune system typically clears most HPV infections within two years; however, persistent infections can progress to more serious conditions like cervical cancer, underscoring the importance of early detection and vaccination to prevent the disease.

Cervical cancer is the fourth most common cancer among women worldwide, with a particularly high burden in low- and middle-income countries due to limited access to vaccination, screening, and treatment services. In Uzbekistan, cervical cancer represents a major public health issue, ranking as the second most frequent cancer among women. The country has 12.2 million women aged 15 and older at risk, with an annual incidence of 1,887 new cases and 1,103 deaths from the disease [1].

Risk factors for HPV infection are multifaceted and include a high number of sexual partners, early sexual activity, smoking, a weakened immune system, opportunistic infections, long-term use of oral contraceptives, and non-condom contraception methods. Other factors such as age, damaged skin, and personal contact with warts or contaminated surfaces also contribute to the risk of contracting HPV [2-5].

The clinical presentation of HPV is often asymptomatic, leading to many infections going undetected until they potentially progress to more serious conditions like cervical cancer [1-3]. This highlights the critical need for effective screening and early detection methods. The traditional method for cervical cancer screening is the Papanicolaou test, also known as the Pap smear test. The Pap smear is good for the early detection of cancer cells; similarly, it is also feasible for detecting HPV deoxyribonucleic acid (DNA) by polymerase chain reaction (PCR), but the collection of samples can be uncomfortable for women and requires specially trained medical staff. Additionally, implementing smear collection for widespread screening presents challenges.

From 2025, Uzbekistan plans to launch a mass screening program for HPV. This study addresses the challenges associated with current screening methods by assessing the reliability of detecting HPV DNA in an alternative method - urine samples. Furthermore, the study included the estimation of risk factors associated with the prevalence of HPV infection, intending to inform future interventions and policies to reduce the burden of HPV-related diseases in Uzbekistan.

## Materials and methods

Characteristics of participants

The study was conducted as a collaborative pilot project between the Research Institute of Virology of Uzbekistan and the Korea Foundation for International Healthcare (KOFIH) in Tashkent, Uzbekistan.

The study carried a cross-sectional design with a control group and was conducted between January and May 2023, and paired (smear and urine) samples were collected from 220 participants. Paired samples were tested by PCR for HPV DNA. The study included women at age ≥ 18, who agreed to be included in the study and signed an informed consent. We excluded pregnant women (postpartum period included), women with tumors of genitalia, and women who had invasive cervical procedures last 6 months. A total of 71 cases were identified as HPV DNA positive by HPV DNA screening of smear samples.

Sample size calculation and sampling

To evaluate the sensitivity and specificity of urine-based HPV DNA testing relative to cervical smear testing, the sample size was determined with the presumption of a 10% HPV infection prevalence rate. An alternative hypothesis was set, suggesting that the urine test could achieve a sensitivity of 80%, against a null hypothesis sensitivity of 50%. This led to the requirement for 200 participants, with an additional 10% included to compensate for any potential loss, resulting in a target sample size of 220 participants [6].

The study began with 71 confirmed HPV DNA-positive cases. To establish a control group, women were randomly selected and invited to participate in the study. This control group consisted of 149 women who had not been vaccinated against HPV, were negative for HPV DNA by cervical smear, and provided informed consent. All participants provided a paired cervical and urine sample. A questionnaire survey was then administered to all participants, including both the HPV-positive cases and the control group, resulting in a total of 220 participants. The questionnaire gathered relevant demographic and health information.

During the study, two participants were excluded due to unclear results from their urine samples. Consequently, the final analysis was conducted on paired urine and cervical smear samples from 218 women. The age range of the final study population was 20 to 52 years.

Collection, transportation, and storage of swab specimen

The study utilized probe type D1 "Cytobrush" (Changzhou Chuangjia Medical Appliances Co.Ltd, Changzhou, China) for the collection of swabs from the vaginal mucous membrane of the cervical canal. The probe was rotated against the lateral walls of the vagina to comprehensively collect the discharge. The probe, with the collected material, was then transferred into a transport medium with mucolytic (TCM-AmpliSens, Moscow, Russia). The working part of the probe containing the specimen was broken off and left in the tube. If it was not feasible to break off the working part, the biomaterial was thoroughly rinsed from the working part into the tube by pressing it against the tube's inner side and rotating it 5-10 times in both directions. The tube was securely sealed to prevent any gap or crushing of the lid's inner part and then labeled. The biological material in the transport medium was stored and transported according to the instructions.

Collection, transportation, and storage of urine samples

A 60 ml midstream portion of the morning urine sample was collected in a sterile container for PCR analysis. The samples were centrifuged at 12,000 revolutions per minute for 15 minutes using a Biosan centrifuge (Biosan SIA, Riga, Latvia), and the supernatant was removed using a non-filtered tip. The remaining cell pellet was then transferred into a transport medium containing mucolytic agents in a 1.5-milliliter tube, mixed using a Microspin FV-2400 vortex (Biosan SIA, Riga, Latvia), and centrifuged for 3-5 seconds at 10,000 revolutions per minute using a laboratory microcentrifuge (Eppendorf, Hamburg, Germany). The urine samples with transport medium were stored at -20 degrees Celsius.

DNA extraction procedure

Following the DNA-sorb-AM instruction manual (AmpliSens), the DNA extraction was conducted by adding 10 microliters of VKO-FL (internal control sample) into sterile polypropylene tubes. The sorbent is then resuspended for 10 seconds using a Microspin FV-2400 vortex (Biosan). This step is followed by the addition of 20 microliters of universal sorbent and 300 microliters of lysing solution (AmpliSens), utilizing aerosol barrier tips for the process. Subsequently, 100 microliters of the urine sample are added to each tube, employing a separate filtered tip for every sample. A negative control is also prepared by adding 100 microliters of a negative control solution into a distinct tube.

After securely closing the tubes, they are thoroughly mixed using the vortex and then incubated for 5 minutes at 65 degrees Celsius in a TDB-120 thermostat (Biosan). Following incubation, the contents are mixed once more on the vortex and allowed to stand at room temperature for 2 minutes. The sorbent is then pelleted by centrifugation at 10,000 revolutions per minute for 30 seconds using an Eppendorf centrifuge, and the supernatant is removed with a vacuum aspirator OM-1, using a separate non-filtered tip for each sample.

Next, 1 milliliter of washing solution is added to each sample, mixed on the vortex until the sorbent is fully resuspended, and centrifuged again under the same conditions. After removing the supernatant, the tubes are placed in a TDB-120 thermostat at 65 degrees Celsius for 5-10 minutes to dry the sorbent with the tube caps open. Then, 100 microliters of Tris-EDTA buffer are added for DNA elution, using a filtered tip. The samples are mixed on the vortex, placed in the thermostat at 65 degrees Celsius for 5 minutes, and centrifuged at 12,000 revolutions per minute for 1 minute (Eppendorf). The supernatant containing purified DNA is then transferred to new tubes as per the manufacturer's instructions.

HPV genotyping by polymerase chain reaction

For the PCR genotyping process, 15 microliters of one of the four prepared reaction mixtures were added to the tubes. Then, 10 microliters of the extracted HPV DNA samples were added to four tubes containing different PCR mixtures. A positive PCR control was prepared by adding 10 microliters of the K2 HPV genotype (weakly positive control of HPV genotype) to four tubes with different PCR mixtures. A negative control for the extraction was prepared by adding 10 microliters of negative control solution to four tubes with different PCR mixtures. Amplification was performed with real-time detection using a Rotor-Gene system (Starlab International GmbH, Hamburg, Germany), and the results were analyzed using the device's software.

Questionnaire survey

After collecting paired cervical and urine samples, all participants underwent an interview using a structured questionnaire. This questionnaire was designed to gather socio-demographic and behavioral data relevant to potential risk factors for HPV infection. The information collected included age, occupation, marital status, number of childbirths, contraceptive methods, number of abortions, age at initiation of sexual activity, date of last cervical cancer screening, and characteristics of genital discharge prior to the test. The discharge characteristics assessed included whether it was foamy, fungal, colorless, had any odor, was purulent, or mucous. Once the results of HPV DNA positivity from cervical smear samples were obtained, participants with positive results were designated as the main group, while those with negative results were assigned to the control group. This grouping facilitated the assessment of factors associated with HPV DNA positivity.

Statistical analysis

Statistical evaluations were conducted using the SPSS software version 29.0 (IBM Corp., Armonk, USA). To determine the level of agreement between urine and cervical sample results, we calculated the agreement rate and kappa coefficient, along with a 95% confidence interval. The diagnostic accuracy of urine-based HPV DNA testing was assessed by its sensitivity, specificity, positive predictive value (PPV), and negative predictive value (NPV), using cervical sample results as a reference.

The univariate and multivariate logistic regression analyses were utilized to assess potential risk factors for HPV infection. The independent variables considered in the analysis included age, occupation, smoking status, marital status, parity, contraceptive use, number of abortions, age at sexual debut, the timing of the last cervical cancer screening, and characteristics of genital discharge prior to testing (such as the presence of foam, fungal elements, colorlessness, odor, purulence, or mucosity). A p-value below 0.05 was deemed to indicate statistical significance.

## Results

Accuracy of urine HPV DNA detection

In the study, DNA was detected in 43 out of 218 urine samples, with two instances of false positives. Additionally, 30 samples that tested positive for HPV through cervical smear tests were not identified as positive by urine PCR testing (Table 1).

**Table 1:** Results of comparison of smear and urine samples by PCR. The detection of DNA from cervical smear was set as a reference test. PCR: polymerase chain reaction

		Cervical smear samples				
		Positive		Negative		Total for urine samples
Urine samples	Positive	41	True positives (TP)	2	False positives (FP)	43
	Negative	30	False negatives (FN)	145	True negatives (TN)	175
Total for cervical samples		71		147		

The study detected high-risk HPV genotypes, which include HPV-16, -18, -31, -33, -35, -39, -45, -51, -52, -56, -58, and -59, in most positive cases: 97.2% of cervical samples with HPV (69/71) and 93.0% of urine samples with HPV (40/43). Additionally, the single low cancerogenic genotype HPV-66 was detected in existing cases. The most frequently occurring HPV type in both sample types was HPV-16, found in 22.5% of HPV-positive cervical samples (16/71) and 25.6% of HPV-positive urine samples (11/43).

Single HPV genotype infection was present in a significant portion of the cervical samples - 66.2% (47/71). In urine samples, single infections were found in 48.8% (21/43) of the cases.

The sensitivity of the urine analysis in comparison with smear analysis demonstrated 57.7%, and specificity of 98.6%. PPV value was 95.3%, with NPV at the level of 82.9% Cohen’s Kappa coefficient was 60.1, which indicates a low border of good agreement (Table 2).

**Table 2:** Accuracy and agreement levels of the urine samples compared with cervical smear samples. TP: true positives; FP: false positives; FN: false negatives; TN: true negatives

Test metrics	Calculation formula	Results
Sensitivity	(TP+FN)/TP	57.7%
Specificity	(TN+FP)/TN	98.6%
Positive predictive value	TP/(TP+FP)	95.3%
Negative predictive value	TN/(TN+FN)	82.9%
Kohen’s kappa		60.1

Factors association assessment of the HPV positivity by cervical smear HPV DNA positivity

Age and HPV DNA Positivity

The analysis revealed a significant association between age and HPV DNA positivity. Participants aged 31-40 years showed a notably higher likelihood of being HPV DNA positive (50.7%), with an odds ratio (OR) of 5.15 (95% CI: 1.12-23.6, p=0.03) in univariate analysis and an adjusted odds ratio (AOR) of 8.2 (95% CI: 1.07-62.4, p=0.04) in multivariate analysis. The group aged ≥41 years also demonstrated an increased association, though not statistically significant in the multivariate analysis (AOR=6.3, 95% CI: 0.82-47.8, p=0.07).

Marital Status

A significant difference in HPV DNA positivity was observed based on marital status in the univariate analysis, with single participants having lower odds of being HPV DNA positive (OR=0.26, 95% CI: 0.04-0.98, p=0.04). However, this association was not significant in the multivariate analysis (AOR=0.32, 95% CI: 0.03-2.86, p=0.31).

Sexual Life, Parity, and Abortions

The initiation of sexual life and the number of parity did not significantly impact HPV DNA positivity in the multivariate analysis. Similarly, having abortions was associated with lower odds of HPV DNA positivity in the univariate analysis (OR=0.52, 95% CI: 0.29-0.95, p=0.03), but this was not significant in the multivariate analysis (AOR=0.51, 95% CI: 0.24-1.09, p=0.08).

Profession Type

Medical staff were found to have a significantly higher risk of HPV DNA positivity (AOR=7.51, 95% CI: 1.61-34.9, p=0.01) compared to other profession types in the multivariate analysis.

Contraception Use

The use of condoms was significantly associated with a lower risk of HPV DNA positivity (AOR=0.12, 95% CI: 0.02-0.64, p=0.01) in the multivariate analysis.

Period from the Last Screening for Cervical Cancer and Vaginal Discharge Characteristics

The last screening for cervical cancer prevention and HPV DNA presence was not associated significantly.

The characteristics of vaginal discharge before screening were analyzed. Notably, foamy (AOR=7.26, 95% CI: 1.1-44.6, p=0.03) and purulent discharge (AOR=6.84, 95% CI: 1.55-30.2, p=0.01) were significantly associated with higher odds of HPV DNA positivity in the multivariate analysis. (Table 3)

**Table 3:** Analysis of factors associated with HPV DNA prevalence in cervical samples through uni- and multivariable logistic regression. R2 (Cox and Snell’s) – 0.316; p<0.001 HPV: human papillomavirus

Variables		Overall paired samples n=218 (100%)	Cervical sample HPV DNA positive – main group n=71 (32.6%)	HPV DNA positivity by cervical smear					
				Univariate analysis			Multivariate analysis		
				OR	[95% CI]	p-value	AOR	[95% CI]	p-value
Age cohorts	≤ 30	18 (8.3%)	2 (2.8%)	1	[Ref.]	-	1	[Ref.]	-
	31-40	97 (44.5%)	36 (50.7%)	5.15	[1.12-23.6]	0.03	8.2	[1.07-62.4]	0.04
	≥ 41	103 (47.2%)	33 (46.5%)	3.71	[0.81-17.1]	0.09	6.3	[0.82-47.8]	0.07
Marital status	Coupled	202 (97.2%)	69 (97.2%)	1	[Ref.]	-	1	[Ref.]	-
	Single	16 (2.8%)	2 (2.8%)	0.26	[0.04-0.98]	0.04	0.32	[0.03-2.86]	0.31
Having a sexual life from age	≤ 20	82 (37.6%)	25 (35.2%)	1	[Ref.]	-	1	[Ref.]	-
	≥ 21	136 (62.4)	46 (64.8%)	1.23	[0.68-2.21]	0.48	1.96	[0.88-4.34]	0.10
Number of parity	0	11 (5.0%)	1 (1.4%)	1	[Ref.]	-	1	[Ref.]	-
	1-3	172 (78.9%)	56 (78.9%)	5.04	[0.63-40.3]	0.12	2.99	[0.22-40.6]	0.41
	≥4	35 (16.1%)	14 (19.7%)	6.67	[0.76-58.1]	0.08	4.13	[0.26-64.7]	0.31
Abortions	No	127 (58.3%)	48 (67.6%)	1	[Ref.]	-	1	[Ref.]	-
	Yes	91 (41.7%)	23 (32.4%)	0.52	[0.29-0.95]	0.03	0.51	[0.24-1.09]	0.08
Profession type	Housewife	155 (71.1%)	53 (74.6%)	1	[Ref.]	-	1	[Ref.]	-
	Medical staff	16 (7.3%)	9 (12.7%)	2.36	[0.83-6.69]	0.10	7.51	[1.61-34.9]	0.01
	Office employee	30 (13.8%)	8 (11.3%)	0.67	[0.28-1.60]	0.36	0.50	[0.14-1.79]	0.29
	Service sector employee	17 (7.8%)	1 (1.4%)	0.12	[0.01-0.89]	0.04	0.11	[0.01-1.25]	0.07
Contraception methods use	No	123 (56.4%)	38 (53.6%)	1	[Ref.]	-	1	[Ref.]	-
	Condoms	30 (13.8%)	3 (4.2%)	0.23	[0.05-0.71]	0.01	0.12	[0.02-0.64]	0.01
	Oral contraceptives	14 (6.4%)	6 (8.4%)	1.57	[0.49-4.83]	0.43	1.29	[0.28-6.02]	0.74
	Intrauterine device (IUD)	51 (23.4%)	24 (33.8%)	1.87	[0.95-3.64]	0.07	1.90	[0.80-4.54]	0.14
The last screening for cervical cancer prevention	0-2 years ago	34 (15.6%)	15 (21.1%)	1	[Ref.]	-	1	[Ref.]	-
	2 and more years ago	184 (84.4%)	56 (78.9%)	0.58	[0.27-1.21]	0.15	0.56	[0.17-1.81]	0.15
Vaginal discharge characteristics before screening registered by gynecologist									
Colorless vaginal discharge	No	54 (24.8%)	8 (11.3%)	1	[Ref.]	-	1	[Ref.]	-
	Yes	164 (75.2%)	63 (88.7%)	3.73	[1.65-8.42]	0.01	4.52	[0.96-21.3]	0.06
Unpleasant odor	No	194 (89%)	57 (80.3%)	1	[Ref.]	-	1	[Ref.]	-
	Yes	24 (11%)	14 (19.7%)	3.22	[1.36-7.89]	0.01	2.43	[0.64-9.18]	0.19
Mucous discharge	No	94 (43.1%)	36 (50.7%)	1	[Ref.]	-	1	[Ref.]	-
	Yes	124 (56.9%)	35 (49.3%)	0.59	[0.33-1.03]	0.07	0.68	[0.19-2.45]	0.56
Foamy discharge	No	200 (91.7%)	56 (78.9%)	1	[Ref.]	-	1	[Ref.]	-
	Yes	18 (8.3%)	15 (21.1%)	12.3	[3.43-44.2]	0.01	7.26	[1.1-44.6]	0.03
Purulent discharge	No	203 (93.1%)	60 (84.5%)	1	[Ref.]	-	1	[Ref.]	-
	Yes	15 (6.9%)	11 (15.5%)	6.29	[1.93-20.5]	0.01	6.84	[1.55-30.2]	0.01

The overall model's goodness of fit, assessed with Cox and Snell’s R², was 0.316, indicating a moderate fit (p<0.001).

Thus, this comprehensive analysis underscores the multifaceted nature of HPV DNA positivity, highlighting the significant roles of age, profession type, contraception use, and characteristics of vaginal discharge.

## Discussion

The results of this study demonstrated that urine HPV DNA testing has good agreement (low border) with к=0.61 and moderate accuracy compared to cervical samples, with a sensitivity of 57.7%, a specificity of 98.6%. The Cohen's kappa coefficient of 60.1 indicates good agreement between the two methods. At this level of sensitivity, the urine method will not detect almost half of positive cases, but we suppose that a high level of specificity is a good alternative to smear samples in mass screening conditions. These findings align with previous studies from various countries showing acceptable concordance between urine and cervical samples for HPV detection [7-10].

The sensitivity of urine HPV testing in this study was lower than some reports from other settings. A meta-analysis by Pathak et al. found a pooled sensitivity of 77% and specificity of 88% for urine HPV testing compared to cervical samples [7]. The next meta-analysis from Bober et al. demonstrated a much higher pooled sensitivity level for any type of HPV infection [11].

The lower sensitivity in the current study may be due to differences in urine collection, in which each woman collected her urine, and the extent to which the urine collection process was professionally explained by medical staff [12, 13].

Several factors were significantly associated with increased odds of HPV positivity in multivariate analysis, including age 31-40 years, working as medical staff, having foamy or purulent vaginal discharge, and not using condoms. Similar risk factors have been reported in other populations, including studies in the Americas, Asia, Europe, and Africa [14-16].

It is important to note that the association between these risk factors and HPV positivity is not isolated from any single demographic or geographic location. For instance, studies have shown that medical staff may have a higher risk of HPV due to increased exposure to the virus in healthcare settings [17]. Vaginal discharge, particularly when foamy or purulent, can be indicative of an underlying HPV infection or other sexually transmitted infections, emphasizing the importance of clinical evaluation and potential screening for HPV in such cases [18].

The use of condoms has been widely advocated as a method to reduce the transmission of sexually transmitted infections, including HPV [19]. The result of our study underscores the importance of comprehensive sexual health education and the promotion of safe sex practices.

The potential of urine HPV testing as a non-invasive screening method is promising despite its relatively low sensitivity, given its high acceptability among women [20]. However, further research is needed to optimize urine collection, storage, and testing protocols to improve sensitivity. Larger, population-based validation studies in Uzbekistan and other countries in Central Asia are warranted to establish the role of urine HPV testing in screening programs.

This study had limitations including the lower sensitivity of urine HPV testing (57.7%) compared to cervical samples, which may result in missed HPV-positive cases. The sample size of 218 participants, while adequate for initial analysis, limits the robustness of the findings, and the exclusion of certain groups (e.g., pregnant women and those with genital tumors) may affect generalizability. Variability in urine collection methods could also impact results. The single-center design restricts the applicability of findings to other regions. Additionally, the cross-sectional nature of the study limits the ability to establish causal relationships between identified risk factors and HPV positivity. Further research with larger, more diverse populations and standardized methodologies is needed to validate and optimize urine-based HPV testing for broader clinical use. 

## Conclusions

The study provides valuable insights into the potential of urine-based HPV testing as a non-invasive screening method, its limitations highlight the need for further research with larger, more diverse populations and standardized methodologies to validate and optimize urine-based HPV testing for broader clinical use. While the study's findings are promising, ongoing efforts to improve screening strategies and their implementation are essential to lessen the impact of cervical cancer in Uzbekistan and worldwide. Collaborative research and the exchange of best practices among countries can expedite progress towards this objective.

The successful implementation of the HPV vaccination program in Uzbekistan (from 2019) highlights the importance of a multi-faceted approach to cervical cancer prevention, combining vaccination, screening, and effective communication strategies.
